# pH-responsive theranostic nanoplatform of ferrite and ceria co-engineered nanoparticles for anti-inflammatory

**DOI:** 10.3389/fbioe.2022.983677

**Published:** 2022-09-09

**Authors:** Yuanyao Dou, Yimin Zhang, Caiyu Lin, Rui Han, Yubo Wang, Di Wu, Jie Zheng, Conghua Lu, Liling Tang, Yong He

**Affiliations:** ^1^ Department of Respiratory Medicine, Daping Hospital, Army Medical University, Chongqing, China; ^2^ Key Laboratory of Biorheological Science and Technology, Ministry of Education, College of Bioengineering, Chongqing University, Chongqing, China

**Keywords:** reactive oxygen species (ROS), Fe/Ce-MSN-PEG NPs, theranostic nanoplatform, anti-oxidative stress, anti-inflammatory

## Abstract

Multiple component integration to achieve both therapy and diagnosis in a single theranostic nanosystem has aroused great research interest in the medical investigator. This study aimed to construct a novel theranostic nanoplatform ferrite and ceria co-engineered mesoporous silica nanoparticles (Fe/Ce-MSN) antioxidant agent though a facile metal Fe/Ce-codoping approach in the MSN framework. The resulted Fe^3+^-incorporated ceria-based MSN nanoparticles possessing a higher Ce^3+^-to-Ce^4+^ ratio than those revealed by ceria-only nanoparticles. The as-prepared Fe/Ce-MSN nanoparticles exhibited an excellent efficiency in scavenging reactive oxygen species (ROS), which is attributed to improving the superoxide dismutase (SOD) mimetics activity by increasing Ce^3+^ content and maintaining a higher activity of catalase (CAT) mimetics *via* including ferrite ion in nanoparticles. The fast Fe/Ce-MSN biodegradation, which is sensitive to the mild acidic microenvironment of inflammation, can accelerate Fe/Ce ion release, and the freed Fe ions enhanced T_2_-weighted magnetic resonance imaging in the inflammation site. PEGylated Fe/Ce-MSN nanoparticles *in vitro* cell models significantly attenuated ROS-induced inflammation, oxidative stress, and apoptosis in macrophages by scavenging overproduced intracellular ROS. More importantly, Fe/Ce-MSN-PEG NPs exhibited significant anti-inflammatory effects by inhibiting lipopolysaccharide (LPS)-induced expression of tumor necrosis factor-α (TNF-α) and interleukin-1 beta (IL-1β) levels *in vitro*. Additionally, it can promote the macrophages polarization of pro-inflammatory M1 phenotype towards an anti-inflammatory M2 phenotype. Thus, the novel pH-responsive theranostic nanoplatform shows great promise for inflammation and oxidative stress-associated disease treatment.

## Introduction

Inflammation is a crucial natural protective response to various inciting stimuli, such as traumatic, infectious, toxic, or autoimmune injury (Nathan, 2002). Aberrant inflammation regulation was regarded as the major cause of human diseases, including diabetes, cardiovascular, neurodegenerative, alcoholic liver, atherosclerosis, pulmonary fibrosis, and cancer (Balkwill and Mantovani, 2001; Wyss-Coray and Mucke, 2002; Biswas and Lopes de Faria, 2006; Libby, 2006; Ambade and Mandrekar, 2012; Li et al., 2015; Wang Y et al., 2018). Generally, the primary process of inflammation is intimately linked to oxidative stress, which is considered an oxidative response to inflammation (Li et al., 2018). The excess production of reactive oxygen species (ROS) was induced by the activated phagocytic cells during an inflammatory response, which causes oxidative damage to cell membranes, proteins, and DNA (Rehman et al., 2012; Nakagawa et al., 2015; Li et al., 2018). Therefore, scavenging excess ROS was considered an anti-oxidative stress strategy for treating various associated diseases with inflammatory disorders. In this aspect, various types of anti-oxidative were developed such as coenzyme, Tempol, vitamins C and E, and NADPH oxidase (Salonen et al., 2003; Wang Y et al., 2018). Unfortunately, undesirable effects were substantiated by preclinical and clinical trials on most antioxidant therapy due to the limited ROS-scavenging capability by a single antioxidant. Thus, a new powerful antioxidant agent remains to be developed.

Researchers in the fields of nanomedicine and bioengineering have produced nanoscale antioxidants with unique ROS-scavenging capability, especially inorganic nanosystems (Martín et al., 2010; Kim et al., 2019). Among all nano-antioxidants, the cerium oxide nanoparticles (CeNPs) were described to act as multi-antioxidants for eliminating ROS based on the multi-enzyme mimetic activity, including superoxide dismutase (SOD) mimetics and catalase (CAT) activity (Carvajal et al., 2019; Ni et al., 2019). Consequently, the beneficial effects of CeNP treatment were exhibited in various injury models, such as the endothelial, neuronal, epithelium, and stem cells (Colon et al., 2010; Mandoli et al., 2010; Chen et al., 2013; Dowding et al., 2014). CeNPs unique antioxidant properties stem from the reversible switching between Ce^3+^ and Ce^4+^ oxidation states depending on the physiological environment (Arya et al., 2013). The Ce^3+^ sites are known to eliminate superoxide radical (O_2_•^-^) *via* SOD-mimetics, and hydroxyl radical (•OH) by redox reactions, whereas the Ce^4+^ sites oxidate hydrogen peroxide (H_2_O_2_) through CAT-mimetics (Pirmohamed et al., 2010). Thus, the ratio of Ce^3+^/Ce^4+^ in CeNPs is important to maintain the multi-enzyme mimetic activity of antioxidant agents. Recent studies revealed that additional CeNP modifications, including lanthanide cation inclusion or transition-metal ion doping, have been developed to improve the antioxidant capabilities (Celardo et al., 2011; Gupta et al., 2016; Zhou et al., 2016; Eriksson et al., 2018). However, few reports have described the diagnostic ability of the modified CeNPs for biomedical applications. Thus, it is urgent to develop a unique CeNP possessing both therapeutic and diagnostic abilities.

**SCHEME 1:**
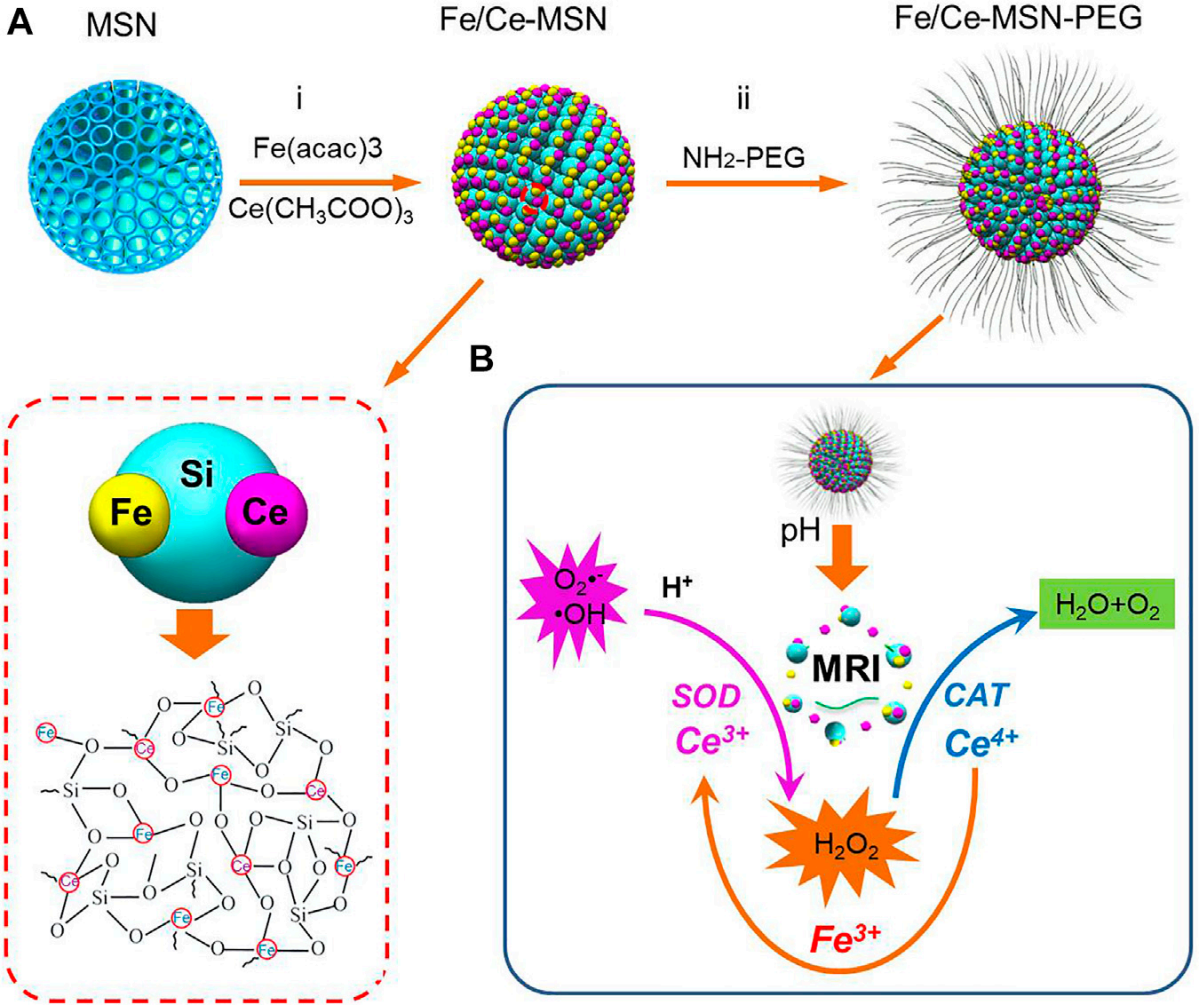
The procedure for the synthesis of Fe/Ce-MSN-PEG NPs is illustrated in **(A)**. **(B)** Schematic illustration of biodegradation, ROS scavenging effects and enhanced theranostic functions by Fe/Ce-MSN-PEG NPs.

This study constructed a novel ferrite and ceria co-engineered mesoporous silica NPs (Fe/Ce-MSNs) antioxidant agent with magnetic resonance imaging (MRI) function for therapeutic agents, where, with the property of scavenging ROS and its potential reaction mechanism in the nanosystem being systematically investigated, these therapeutic agents can efficiently scavenge ROS and exert anti-inflammatory effects (Scheme 1). The excellent efficiency of scavenging ROS attributed to the improved SOD-mimetics activity by increased Ce^3+^ content and maintaining a higher CAT-mimetic activity by including ferrite ion in nanoparticles. Meanwhile Fe/Ce-MSN NPs exhibits the MRI performance and pH stimuli-responsive biodegradability. This as-prepared antioxidant agent was used to attenuate oxidative stress-induced inflammation and apoptosis in anti-inflammatory macrophages by effectively eliminating multiple species of reactive oxygen.

## Materials and methods

### Materials

Tetraethyl orthosilicate (TEOS), Cetyltrimethylammonium bromide (CTAB) were obtained from Sinopharm Chemical Reagent Co. Urea, ferric acetylacetonate (Fe(acac)_3_), fluorescein isothiocyanate (FITC), Cerium (III) acetate hydrate, anhydrous dimethylsulfoxide (DMSO), 2′,7′-Dichlorofluorescin diacetate (DCF-DA) and 2,2-diphenyl-1-picrylhydrazyl (DPPH) were purchased from Sigma-Aldrich (USA). mPEG-NH2 (5kD) was purchased from Aladdin (China). Sodium salicylate (NaSal), triethanolamine (TEA), Cell Counting Kit-8 (CCK-8) were purchased from MedChemExpress (MCE, USA). Penicillin, streptomycin, fetal bovine serum (FBS), and Dulbecco’s Modified Eagle’s Medium (DMEM) were purchased from Hyclone (USA). 4′, 6-Diamidino-2-phenylindole (DAPI), Lyso-Tracker Red, total superoxide dismutase assay kit with WST-8, and catalase activity assay kit were purchased from Solarbio (China). CD68 antibody, Alexa Fluor 549 anti-Nos2 (iNOS) antibody, and CD206 antibody were purchased from Biolegend (USA). Tumor necrosis factor-α (TNF-α), interleukin-1β (IL-1β) ELISA kit were purchased from FineTest (China).

### Synthesis of Fe/Ce co-doped mesoporous silica NPs (Fe/Ce-MSNs)

#### Synthesis of mesoporous silica nanoparticles (MSNs)

MSN NPs were prepared as reported (Gao et al., 2019). In a typical protocol, TEA (0.068 g) was diluted in pure water (25 ml) at 80°C under magnetic stirring for 0.5 h, followed by adding NaSal (84 mg) and CTAB (380 mg) (the CTAB/NaSal molar ratio is 0.5: 1) to the reaction solution and keeping stirring for another 1 h. Then, TEOS (4 ml) was added dropwise into the reaction solution with gentle stirring for 2 h. The products were collected by high-speed centrifugation (20,000 rpm, 20 min at room temperature) and washed several times with water and ethanol to remove the residual reactants. Finally, the obtained products were extracted with HCl and ethanol solution (HCl: ethanol = 1:9) at 80°C (6 h, three times) to remove the surfactants (CTAB) and dried in vacuum at room temperature overnight.

#### Synthesis of Fe/Ce co-doped mesoporous silica NPs (Fe/Ce-MSNs)

The synthesis protocol was modified from the published work (Wang L et al., 2018). In brief, MSNs (25 mg), urea (13.5 mg), Ce(CH_3_COO)_3_ (5 mg) and Fe(acac)3 (10 mg) (the Ce/Fe molar ratio is bout 1:2) were dissolved into a mixture solution containing deionized water (7.5 ml) and ethanol (5 ml). The system was allowed to homogenize under sonication for several minutes before it was transferred and sealed into the autoclave (30 ml). Then the autoclave was placed into an oven and heated to 200°C for 2 h. Fe/Ce-MSNs nanospheres were formed after rinsing with ethanol and water.

The synthesis of Fe-MSNs and Ce-MSNs is similar to that of Fe/Ce-MSNs.

### PEGylation of Fe/Ce co-doped mesoporous silica NPs (Fe/Ce-MSN-PEGs)

Typically, Fe/Ce-MSN NPs (10 mg) and mPEG-NH_2_ (5 mg) were dispersed in anhydrous ethanol (10 ml) and stirred for 24 h. After collected by centrifugation and washed with ethanol and deionized water for several times, the PEGylated Fe/Ce-MSN NPs were obtained.

The synthesis of Fe-MSN-PEG and Ce-MSN-PEG is similar to that of Fe/Ce-MSN-PEGs.

### Characterization of Fe/Ce-MSN nanoparticles

The morphology of Fe/Ce-MSNs was observed with transmission electron microscopy (TEM) (TECNAI-10 microscope, Philips, Netherlands) and Scanning electron microscopy (SEM) (FIB-SEM microscope, Crossbeam 340, Zeiss). Energy dispersive X-ray spectroscopy (EDS) was performed simultaneously on JEM-2100F electron microscope. The phases of Fe/Ce-MSNs were determined by X-ray diffraction (XRD Bruker D8 Focus) with a monochromatized source of Cu Ka1 radiation (l = 0.15405 nm) at 1.6 kW (40 kV, 40 mA). X-ray photoelectron spectroscopy (XPS) was acquired to observe the different valence states of doped ferrite and ceria component on an ESCALAB 250Xi X-ray photoelectron spectroscopy. Dynamic light scattering (DLS) was measured to determine the hydrodynamic particle sizes of PEGylated Fe/Ce-MSNs on a Zetasizer Nanoseries (Nano ZS90). The generated oxygen (O_2_) was measured by an oxygen-sensitive electrode on Multi-Parameter Analyzer (JPSJ-606 L, Leici China).

### ROS-scavenging capability by Fe/Ce-MSNs

#### Elimination of radical by Fe/Ce-MSNs and Fe/Ce-MSN-PEGs

The free radical scavenging capability of Fe/Ce-MSNs and Fe/Ce-MSN-PEGs were measured using a previously established protocol with minor modifications. Briefly, 1.0 ml of a fresh solution of DPPH (100 μg/ml) was incubated in 3 ml of methanol containing different concentrations of Fe/Ce-MSNs and Fe/Ce-MSN-PEGs (From 0.05, 0.1, 0.2, 0.5, to 1.0 mg/ml) for 30 min in dark. Subsequently, the absorbance at 517 nm was recorded by UV-visible spectroscopy (Cary 60 UV-Vis, Agilent) and the DPPH elimination rates were calculated.

#### Scavenging of superoxide radical (O_2_•^-^), hydroxyl radical (•OH) and hydrogen peroxide (H_2_O_2_) by Fe/Ce-MSNs

To examine the superoxide anion-eliminating capability of Fe/Ce-MSNs, different concentrations of Fe/Ce-MSNs (From 0.05, 0.1, 0.2, 0.5, to 1.0 mg/ml) were incubated with an excess amount of superoxide anion. The remaining superoxide anion was measured by the Superoxide Anion Free Radical Detection Kit (Nanjing Jiancheng Bioengineering Institute, China). Of note, the superoxide anion was generated by the reaction of xanthine with xanthine oxidase. Finally, the superoxide anion-eliminating capacity of Fe/Ce-MSNs was quantified by measuring the absorbance at 550 nm.

The hydroxyl radical scavenging capability of Fe/Ce-MSNs (From 0.05, 0.1, 0.2, 0.5, to 1.0 mg/ml) was evaluated. Using the Hydroxyl Radical Detection Kit (Nanjing Jiancheng Bioengineering Institute, China), the hydroxyl radical-eliminating capacity of our nanoparticles was quantified by measuring the absorbance at 550 nm.

In addition, the hydrogen peroxide scavenging capability of Fe/Ce-MSNs was evaluated. In separate experiments, various concentrations of Fe/Ce-MSNs (From 0.05, 0.1, 0.2, 0.5, to 1.0 mg/ml) were incubated in 2 ml of PBS containing 200 µM H_2_O_2_ at 37°C for 24 h. Using the Hydrogen Peroxide Detection Kit (Nanjing Jiancheng Bioengineering Institute, China), the concentration of remaining H_2_O_2_ was determined by measuring the absorbance at 405 nm, and the H_2_O_2_-eliminating capacity was calculated.

### SOD and CAT-mimetic activity of Fe/Ce-MSNs

#### SOD-mimetic activity and kinetic assay

SOD-mimetic activity of Fe-MSN, Ce-MSN, and Fe/Ce-MSN NPs were investigated using the total Superoxide Dismutase Assay Kit with WST-8 as following the provided protocol. Briefly, WST-8 could react with superoxide radical anion (O_2_•^-^) generated by xanthine oxidase to produce the water-soluble formazan dye. The reaction could be blocked by SOD due to the SOD could catalyze the dismutation of O_2_•^-^ to produce hydrogen peroxide (H_2_O_2_) and O_2_. Therefore, the production of formazan dye was negatively correlated with the SOD activity. After incubation with 0.5 mg/ml of nanoparticle, the absorbance was measured at 450 nm. One unit of SOD activity was defined as the amount of enzyme to reduce 50% of WST-8 formazan formation. The kinetic assays of Fe/Ce-MSN and Fe/Ce-MSN-PEG NPs with various concentrations (0.015, 0.03, 0.06, 0.125, 0.25, 0.5, 1.0 mg/ml) were preformed, respectively. The Michaelis Menten constant was calculated according to the Michaelis Menten saturation curve in Origin 9.

#### CAT-mimetic activity and kinetic assay

The catalase (CAT) activities of Fe-MSN, Ce-MSN and Fe/Ce-MSN NPs were investigated using a catalase activity assay kit, by measuring the peroxidase activity. The nanoparticle suspension was mixed with reaction buffer, followed by H_2_O_2_ addition. The decomposition of H_2_O_2_ was terminated by adding stop solution and the remaining H_2_O_2_ was determined by using peroxidase. This was based on the formation of (N-(4-antipyryl)-3-chloro-5-sulfonatepbenzoquinonemonoimine), which was measured at a wavelength of 520 nm. One unit of CAT activity was defined as the amount of enzyme that consumed 1 mM H_2_O_2_ per minute at 25°C and pH 7.4.

We evaluated the CAT kinetic parameters of Fe/Ce-MSN and Fe/Ce-MSN PEG NPs at a fixed concentration by measuring the generated oxygen using a dissolved oxygen meter. The kinetic assays of Fe/Ce-MSN and Fe/Ce-MSN PEG NPs (0.5 mg/ml) at pH 7.4 with H_2_O_2_ (20, 50, 100, 200, and 400 mM) were performed at 37°C, respectively. The *K*_*m*_ and *V*_max_ were calculated by using Michaelis Menten saturation curve in Origin 9.

### Degradation experiment of Fe/Ce-MSN nanoparticles *in vitro*

*In vitro* degradation profiles and microstructure evolutions of Fe/Ce-MSN NPs were assessed by two typical approaches. One was detecting the degradation content of Fe, Ce and Si by inductively coupled plasma mass spectrometry (ICP-MS, 7800 ICP-MS, Agilent), and the other was directly observing the time-dependent structural evolution of Fe/Ce-MSNs by SEM during the degradation evaluation.

Typically, 1 mg Fe/Ce-MSNs was added into 1 ml FBS (PBS as control) solution with different pHs (pH: 7.4 and 6.0) for 24 h. The testing solution was put into a water bath at 37°C under magnetic stirring slowly (250 rpm). ICP-MS test and SEM images of samples were taken out at different time points to monitor the degradation.

### *In vitro* MRI assay

The *in vitro* MR imaging experiments were performed on a 3.0 T clinical MRI instrument (GE Signa LX 3.0 T). The manganese concentrations of Fe/Ce-MSNs dispersed in FBS solution with different pHs (pH = 7.4 and 6.0) were determined by ICP-MS after incubation 24 h. T_2_-weighted Fast-recovery spin-echo (FR-FSE) sequence is described as follows: TR = 1000, 2000, 3000 and 4000, Slice = 3, Space = 0.5 mm, Fov = 20, Phase fov = 0.8, Freq x Phase = 384 x 256, Nex = 2, ETL = 2.

### Cytotoxicity evaluation by CCK-8 assay

A RAW264.7 mouse macrophage cells and 3T3 fibroblasts were cultured in 96-well plates at a density of 1.0 × 10^4^ cells per well in 100 µL DMEM containing 10% (v/v) FBS, 100 U/ml of penicillin, and 100 mg/ml of streptomycin. Cells were incubated at 37°C in a humidified atmosphere containing 5% CO_2_ before additional experiments. After 24 h, cells were treated with Fe-MSN-PEG, Ce-MSN-PEG, and Fe/Ce-MSN-PEG NPs (from 0.02, 0.05, 0.1, 0.2, 0.5, 1 and 2 mg/ml). The cell viability was quantified by CCK-8 assay.

### Cellular uptake studies

The nanoparticles were labeled with FITC before cellular uptake assay according to the previous report (Chen et al., 2012). Briefly, 15 mg of fluorescein isothiocyanate (FITC) was reacted with 100 ml 3-aminopropyltriethoxysilane (APTES) in 5 ml of absolute ethanol under dark conditions for 24 h. Subsequently, 20 mg of Fe/Ce-MSN-PEG NPs (or Fe-MSN-PEG, Ce-MSN-PEG) were reacted with FITC-APTES stock solution (1 ml) under dark conditions for 24 h. The FITC grafted nanoparticles were collected by centrifugation and washed with ethanol several times to remove the unreacted FITC-APTES. The product was finally dried under vacuum at room temperature in the dark.

Cellular uptake assay: RAW264.7 mouse macrophage cells were seeded at a density of 2.0 × 10^5^ cells per well into a confocal dish and incubated overnight. Then, cells were treated with FITC loaded Fe-MSN-PEG, Ce-MSN-PEG and Fe/Ce-MSN-PEG NPs respectively for 4 h. At the end of treatment, cells were washed with PBS 3 times, then, stained with DAPI and Lyso-Tracker Red, and fixed with methanol. The fluorescence images were acquired by confocal laser scanning microscopy (Olympus, Japan, FV1000).

### *In vitro* anti-apoptosis activity of Fe/Ce-MSN-PEG NPs in macrophage cells

RAW264.7 mouse macrophage cells were seeded in a 12-well plate at a density of 3 × 10^5^ cells/well and incubated overnight. Cells were incubated with Fe-MSN-PEG, Ce-MSN-PEG, and Fe/Ce-MSN-PEG NPs respectively at 0.5 mg/ml for 2 h, and then the culture medium was replaced with 1 ml of fresh medium containing 200 μM H_2_O_2_. After 24 h of incubation, cells were washed with a cold BioLegend’s cell staining buffer, collected by centrifugation, and resuspended in an Annexin V binding buffer containing Annexin V-APC (Annexin V) and propidium iodide (PI) solution. After cells were vortexed gently and incubated in a dark room for 15 min, flow cytometry analysis (Beckman: Cytomics FC 500, Cyt, USA) was performed immediately.

### Intracellular Scavenging of ROS in macrophage cells

RAW264.7 mouse macrophage cells were cultured in 12-well plates for 12 h. After cells were pretreated with various doses of Fe-MSN-PEG and Ce-MSN-PEG and Fe/Ce-MSN-PEG NPs (0.5 mg/ml) respectively for 2 h, they were stimulated with 200 µM H_2_O_2_ for 24 h. The normal control group was treated with fresh medium, and the model group was stimulated with H_2_O_2_ without treatment with nanoparticles for 24 h. Then, a final concentration of 10 µM of DCF-DA in serum-free medium was added to the cells and incubated in dark at 37°C for 30 min. Afterwards, the cells were washed with serum-free medium thrice to remove unloaded DCF-DA probe, then were imaged using a fluorescence microscope (Leica DM2500, Germany) and were subjected to flow cytometry analysis to quantify the intracellular ROS levels respectively.

### *In vitro* anti-inflammation effects of Fe/Ce-MSN-PEG NPs in macrophages

Specifically, RAW264.7 macrophages were seeded in 6-well plates at a density of 4 × 10^5^ cells/well and incubated overnight. After co-culture with Fe-MSN-PEG, Ce-MSN-PEG and Fe/Ce-MSN-PEG NPs (0.5 mg/ml) respectively for 4 h, cells were stimulated with LPS (100 ng/ml) for another 6 h. Subsequently, typical inflammatory cytokines in the culture supernatant, including tumor necrosis factor-α (TNF-α), interleukin-1β (IL-1β) were determined by ELISA.

### Macrophage polarization

RAW264.7 macrophages were stimulated in medium containing 200 ng/ml LPS for 24 h. Then cells were treated with different formulations for 8 h. Subsequently, macrophages were labeled with different combinations of antibodies to identify M1 and M2 macrophages. Cy5.5-conjugated rat anti-mouse CD68 antibody and Alexa Fluor 549 anti-Nos2 (iNOS) antibody were used for staining M1 macrophages, while M2-like macrophages were labeled with Cy5.5-conjugated rat anti-mouse CD68 antibody and PE-conjugated rat anti-mouse CD206 antibody. The corresponding isotype controls were also used. After washing twice with PBS, samples were analyzed by flow cytometry.

### Statistical analysis

Data are expressed as mean ± standard error of the mean (SE). Statistical analysis was assessed using One-way ANOVA test. A value of *p* < 0.05 was considered statistically significant.

## Results

### Synthesis and characterization of Fe/Ce-MSN-PEG NPs

The procedure for Fe/Ce-MSN-PEG NP synthesis is illustrated in Scheme 1A. Firstly, MSNs with unique central-radial pore structures were synthesized by hydrolyzation of tetraethyl orthosilicate based on the StÖer mechanism and sol-gel chemistry, and then immediately utilized as the hard template for metal ion doping (Yang et al., 2016). Secondly, with the inner part of MSNs being dissolved under a high-temperature hydrothermal reaction to release silica oligomers, the Fe and Ce ions engineering into the framework of MSNs was produced by *in situ* topological (Wang L et al., 2018). Meanwhile, Fe and Ce ions could self-assemble with the released silica oligomers to restructure the framework of MSNs after the addition of ferric and cerium-containing precursors into the system (names as Fe/Ce-MSNs). Finally, Fe/Ce-MSNs were modified with polyethylene glycol (PEG) through a layer-by-layer polymer-coating technology to enhance their water solubility and physiological stability (Scheme 1A) (Karakoti et al., 2009).

**FIGURE 1:**
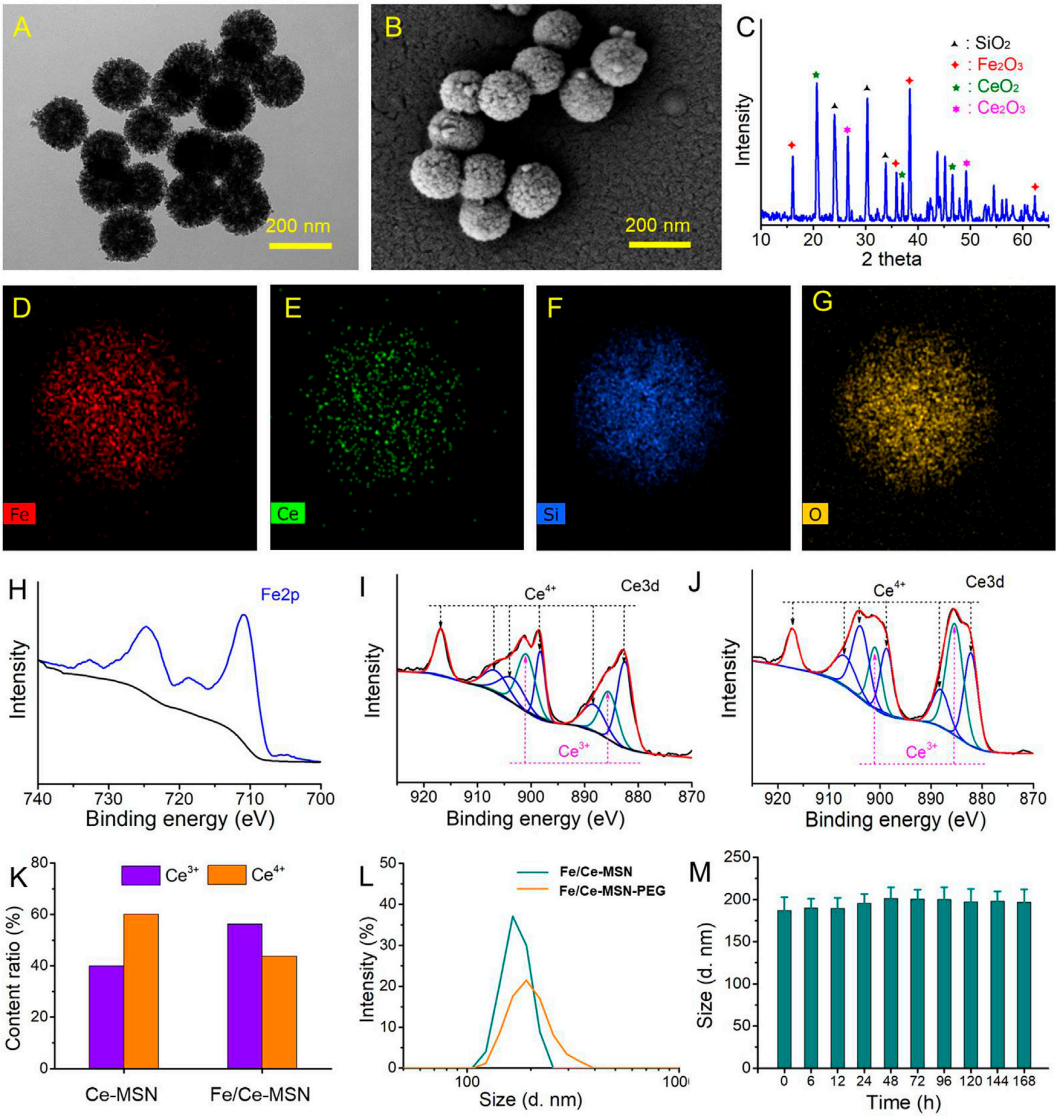
Morphological and compositional characterizations of Fe/Ce-MSN NPs: **(A–C)** TEM, SEM images and XRD patterns of Fe/Ce-MSN NPs. **(D–G)** Elemental mapping of Fe/Ce-MSN: Fe, Ce, Si, and O. h) XPS spectra of Fe 2p. **(I,J)** XPS spectra of Ce 3d before and after introduce Fe ions. **(K)** The content ratio of Ce^3+^ and Ce^4+^ from the XPS spectra in **(I,J)**. **(L)** Typical particle size distribution diagrams of Fe/Ce-MSN and Fe/Ce-MSN-PEG NPs. **(M)** the physical stability of Fe/Ce-MSN-PEG NPs at 37°C in serum (5%, pH 7.4). The hydrodynamic size of Fe/Ce-MSN-PEG NPs was determined over 1 week.

Transmission electron microscopic (TEM) images showed that Fe/Ce-MSN nanoparticles exhibit unique loose porous nanostructures with a uniform diameter of approximately 160 nm (Figure 1A, the TEM images of MSNs, Fe-MSNs and Ce-MSNs see Supplementary Figures S1A–C). Scanning electron microscope (SEM) patterns of as-prepared Fe/Ce-MSN NPs exhibit a mesoporous spherical morphology with a rough surface (Figure 1B). The X-ray diffraction (XRD) pattern (Figure 1C) of the as-prepared sample shows several peaks of Fe_2_O_3_, Ce_2_O_3,_ and CeO_2_. The energy dispersive X-ray spectroscopy (EDX) elemental mapping (Figures 1D–G) demonstrated the presence of Fe, Ce, Si, and O ions in the whole matrix of Fe/Ce-MSN, which illustrates a considerable quantity of doped Fe and Ce in the MSN framework. The Fe and Ce ions loading amount was measured as approximately 20.12% and 9.94% (w/w) in Fe/Ce-MSN NPs, respectively, *via* inductively coupled plasma mass spectrometry (ICP-MS). X-ray photoelectron spectroscopy (XPS) analysis was applied to further explore the elemental composition and valence state of the as-prepared nanoparticles (Figures 1H–J). The two characteristic peaks of 710.8 and 724.5 eV in the Fe 2p spectrum (Figure 1H) were assigned to Fe 2p_3/2_ and Fe 2p_1/2_, respectively, and were accompanied by two weak satellite peaks of approximately 718.7 and 732.5 eV, indicating the Fe^3+^ oxidation state of Fe species in our products (Zhang et al., 2010). As shown in Figures 1I,J, Ce three-dimensional spectrum showed the binding energy levels of Ce^3+^ (885.0 and 903.5 eV) and Ce^4+^ (882.1, 888.1, 898.0, 900.9, 906.4, and 916.4 eV), according to representation method exhibited in the literature, (Ni et al., 2019; Qin et al., 2019). Furthermore, the synthesized Fe/Ce-MSN NPs contained approximately 56.3% of cerium (III) oxides, which is higher than that of doped Ce alone in MSN particles (≈40.0% of cerium (III) oxides in Ce-MSN NPs as shown in Figures 1I–K). The presence of Ce^3+^ benefits the Ce^4+^/Ce^3+^ redox cyclic process on wheels because the redox potential of the Ce^4+^/Ce^3^ redox pair was reduced in rich Ce^3+^ content conditions (Zhang et al., 2018). We used PEGylation modified method to improve the NP water-dispersible because as-synthesized Fe/Ce-MSN NPs are hydrophobic (Karakoti et al., 2009). The hydrodynamic size was increased from 165 ± 2.3 nm of Fe/Ce-MSNs to 190 ± 1.2 nm of PEGylation modified NPs, as seen in Figure 1L. Additionally, the long-term stability of Fe/Ce-MSN-PEG NPs at 37°C in 5% serum (pH: 7.4) can be estimated by assessing the nanoparticle size fluctuation (Figure 1M). The average size of Fe/Ce-MSN-PEG NPs fluctuated in a small range within 7 days, illustrating no obvious aggregation. Therefore, we can hypothesize that the requirements for enhancing ROS-scavenging activity are successfully met by our Fe/Ce-MSN-PEG NPs.

### Characterization of the ROS-scavenging capability of Fe/Ce-MSN NPs

**FIGURE 2:**
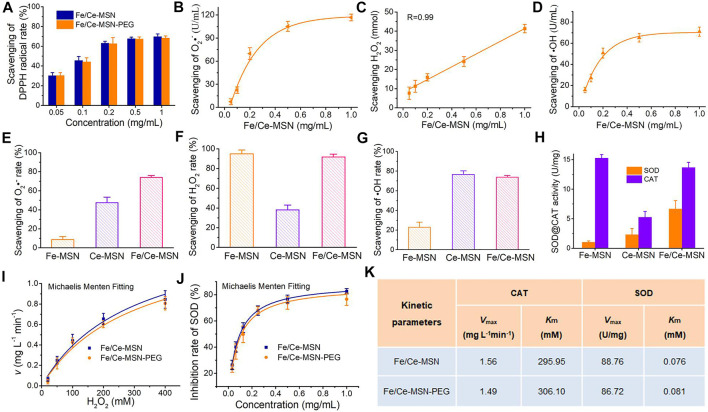
ROS-scavenging capabilities of Fe/Ce-MSN NPs. **(A)** Doses-dependent scavenging of the DPPH radical Fe/Ce-MSN and Fe/Ce-MSN-PEG NPs (*n* = 3). **(B–D)** Elimination of superoxide anion, H_2_O_2_ and hydroxyl radical by Fe/Ce-MSN NPs after incubation 8 h. **(E–G)** Comparison of scavenging capabilities of different materials for radical, superoxide anion, H_2_O_2_ and hydroxyl radical. **(H)** SOD or CAT activity of Fe-MSN, Ce-MSN and Fe/Ce-MSN NPs. Enzyme kinetics of Fe/Ce-MSN and Fe/Ce-MSN-PEG NPs. **(I,J)** Michaelis Menten kinetic analysis of CAT and SOD activity. **(K)** Comparison of the kinetic parameters of CAT and SOD activity, including the maximum reaction velocity (*V*_max_) and Michaelis Menten constants (*K*_m_), in which *K*_m_ confirms the affinity of the nanozyme to the substrate (low *K*_m_ indicates a high affinity) and *V*_max_ represents the catalytic activity of the nanozyme (Dong et al., 2022).

The ROS-scavenging ability of Fe/Ce-MSN NPs was investigated to evaluate its potential antioxidant action. First, the free radical eliminating capability of Fe/Ce-MSN and Fe/Ce-MSN-PEG NPs were compared by 2,2-diphenyl-1- picrylhydrazyl (DPPH) assay because this free radical model is stable enough for laboratory assays of the compounds’ radical-scavenging activity (Xie et al., 2019). The DPPH radical-scavenging ability of these two NPs presented a concentration-dependent manner as shown in Figure 2A, where the radical-scavenging ratio is proportional to Fe/Ce-MSN and Fe/Ce-MSN-PEG NP doses. Typically, 2 h incubation led to approximately 70% scavenging of the DPPH radical at 1.0 mg/ml of Fe/Ce-MSN NPs. Meanwhile, the ROS-scavenging activity of Fe/Ce-MSN-PEG NPs is slightly inferior to that of Fe/Ce-MSN, indicating the effect of PEG on ROS scavenging is negligible.

Then, typical biologically relevant ROS species, superoxide radical (O_2_•^-^), hydroxyl radical (•OH), and hydrogen peroxide (H_2_O_2_) (Wang and Jiao, 2000) being selected to investigate the ROS-scavenging activity of Fe/Ce-MSN NPs at different concentrations. As expected, our co-doping material was able to effectively scavenge O_2_•^-^, H_2_O_2_, and •OH species as shown in a dose-response pattern after incubation for 8 h (Figures 2B–D). Scavenge rates of O_2_•^−^, H_2_O_2_, and •OH are 73.9%, 95.6%, and 70.5%, respectively, at 1 mgml^−1^ of Fe/Ce-MSN NPs. Only Fe/Ce-MSN NPs can efficiently scavenge all the examined ROS compared with Fe-MSN and Ce-MSN NPs (Figures 2E–G). Therefore, we conclude that the excellent ROS-scavenging effect of our Fe/Ce-MSN NPs is attributed to the Fe-doping into Ce-MSN.

Ceria NPs, as the antioxidant, are reported due to the robust multiple ROS-scavenging capabilities based on mimic enzyme properties of CAT-mimetics, accounting for the cerium (IV) sites and SOD-mimetics, attributing to the cerium (III) sites (Korsvik et al., 2007; Pirmohamed et al., 2010; Ni et al., 2019). This study conducted such mimic enzyme activity of Fe-MSN, Ce-MSN, and Fe/Ce-MSN NPs. The SOD activity of Fe/Ce-MSN NPs increased to 6.7 U/mg after the Fe ion was incorporated into Ce-MSN (SOD activity of 2.3 U/mg), and the CAT activity increased to 13.6 U/mg. This result can be explained by the rise of the Ce^3+^/Ce^4+^ ratio in nanoparticles after the Fe ions were introduced based on the XPS results (Gupta et al., 2016). The improved Fe/Ce-MSN ability to scavenge superoxide and hydroxyl radicals is attributed to higher SOD enzyme activity. Additionally, the enhanced hydrogen peroxide consumption capacity is explained by two aspects: maintains a higher CAT enzyme activity and introduces catalysis effect of Fe_2_O_3_ *via* the inclusion of ferrite ion in nanoparticles (Wang L et al., 2018). Therefore, such mimic enzyme activity is critical to improve the different ROS-scavenging capabilities. These results support the hypothesis that a higher Ce^3+^-to-Ce^4+^ ratio, by facilitating the Fe ion incorporation, is the key to the O_2_•^−^ and •OH reduction. Notably, in all these assays, the Fe/Ce-MSN NPs exhibit the best ROS-scavenging performance, indicating that Fe ion introduction into ceria nanoparticles greatly improves the ROS-scavenging activity.

To further reveal the CAT-like enzymatic catalysis mechanism, a steady-state kinetic analysis was performed by changing the concentration of H_2_O_2_ (20, 50, 100, 200, and 400 mm) for O_2_ generation at a fixed concentration of Fe/Ce-MSN and Fe/Ce-MSN-PEG NPs (Dong et al., 2022), which was consistent with the classic Michaelis Menten kinetics (Figure 2I). The values of *K*_m_ and *V*_max_ were solved to be 295.95 mM and 1.56 mg L^−1^ min^−1^ for the Fe/Ce-MSN NPs, whereas those of Fe/Ce-MSN-PEG NPs were 306.1 mM and 1.49 mgl^−1^ min^−1^ (Figure 2K). We also compared the kinetic parameters of SOD-like enzymatic in Fe/Ce-MSN and Fe/Ce-MSN-PEG NPs. The inhibition rate of SOD enzyme by different concentration of these was measured, and the specific activity was calculated to find out the Michaelis Menten kinetics (Figure 2J). These two nanoparticles displayed similar *K*_m_ and *V*_max_ values as seen in Figure 2K. Therefore, the above results indicated that the effect of PEG on the kinetic parameters of SOD and CAT for Fe/Ce-MSN and Fe/Ce-MSN-PEG NPs is negligible.

### Biodegradation study of Fe/Ce-MSN NPs sensitive to acidic microenvironment

**FIGURE 3:**
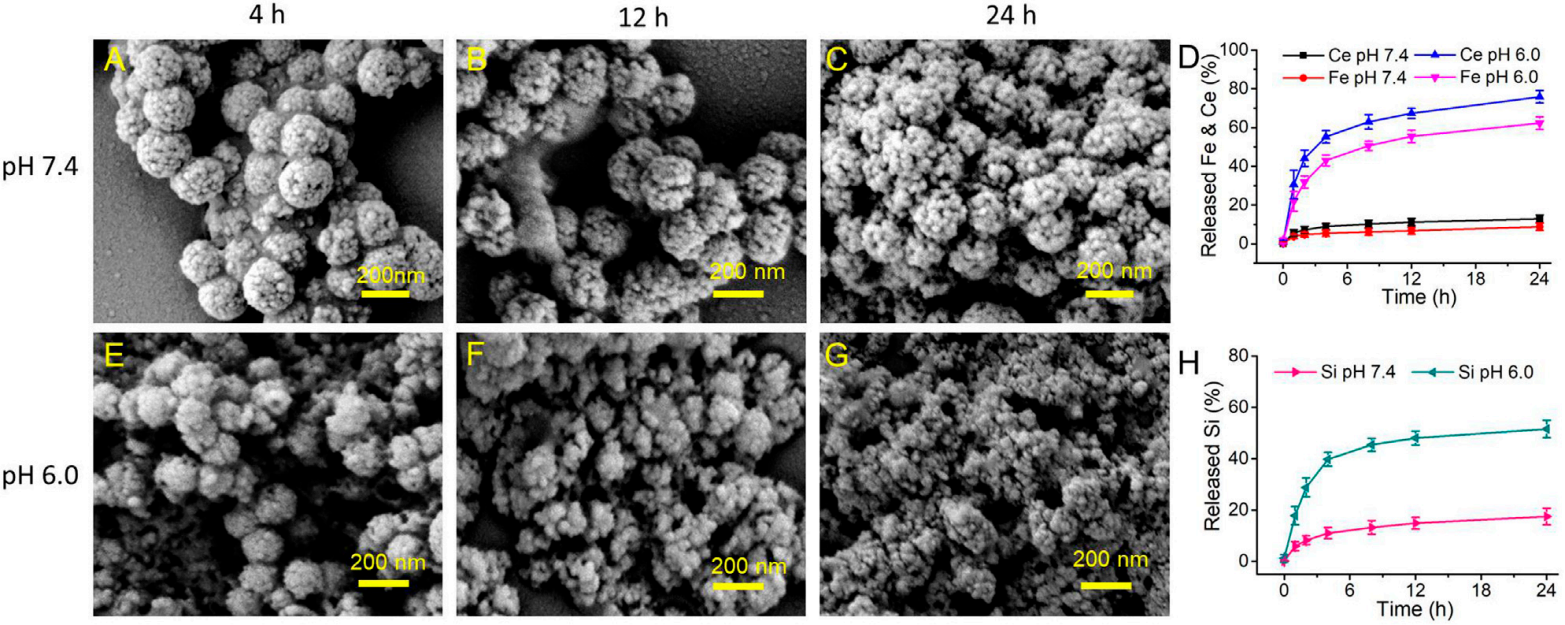
Decomposition of Fe/Ce-MSN NPs: **(A–C)** SEM images of Fe/Ce-MSN NPs after incubation in FBS solution at pH 7.4 for various periods of time, and **(E–G)** SEM images of Fe/Ce-MSN NPs after incubation in FBS solution at pH 6.0 for various periods of time. **(D,H)** Accumulated releasing profiles of Ce, Fe and Si elements in FBS solution under at different pH (7.4 and 6.0).

Introduction of metal ion bonds into the -Si-O-Si- framework of MSNs could alter the biodegradation nature of MSNs, and more importantly, make the degradation sensitive to the special acidic microenvironment (Yu et al., 2016). Additionally, the incorporation of iron (III) into the silica framework has been well-demonstrated to promote the silica matrix degradation in human and fetal bovine serum (FBS) based on the strong coordination interaction between proteins and Fe ions (Peng et al., 2013; Wang L et al., 2018). Herein, we have explored the Fe/Ce-MSN NPs biodegradation in FBS under the varied pHs (7.4 and 6.0). The as-prepared Fe/Ce-MSN NPs were directly immersed into SBF solutions with different pHs, and the degradation process was monitored by SEM observations and ICP-MS tests for various treated time (Figure 3). The morphology of Fe/Ce-MSN NPs showed no significant change in pH of 7.4 conditions after 24 h (Figures 3A–C), indicating the stability of our products in the neutral environment. Surprisingly, Fe/Ce-MSN NPs exhibited time-dependent degradation behavior in pH of 6.0 solution due to the interaction between proteins and Fe ions (Yu et al., 2016) (Figures 3E–G). The release of Fe ions from Fe/Ce-MSN NPs is substantially accelerated under mildly acidic conditions (pH: 6.0) compared to that in neutral FBS solution, which could promote the release of Ce ions (Figure 3D). The SEM images and Fe/Ce releasing profiles provide direct evidence that the break-up of Fe-O and Ce-O bonds in acidic conditions promotes the fast biodegradation of -Si-O-Si- bonds afterward, thus further demonstrating the ultrasensitive pH-responsive degradation behavior of Fe/Ce-MSN NPs.

### Magnetic resonance imaging performance of Fe/Ce-MSN NPs

**FIGURE 4:**
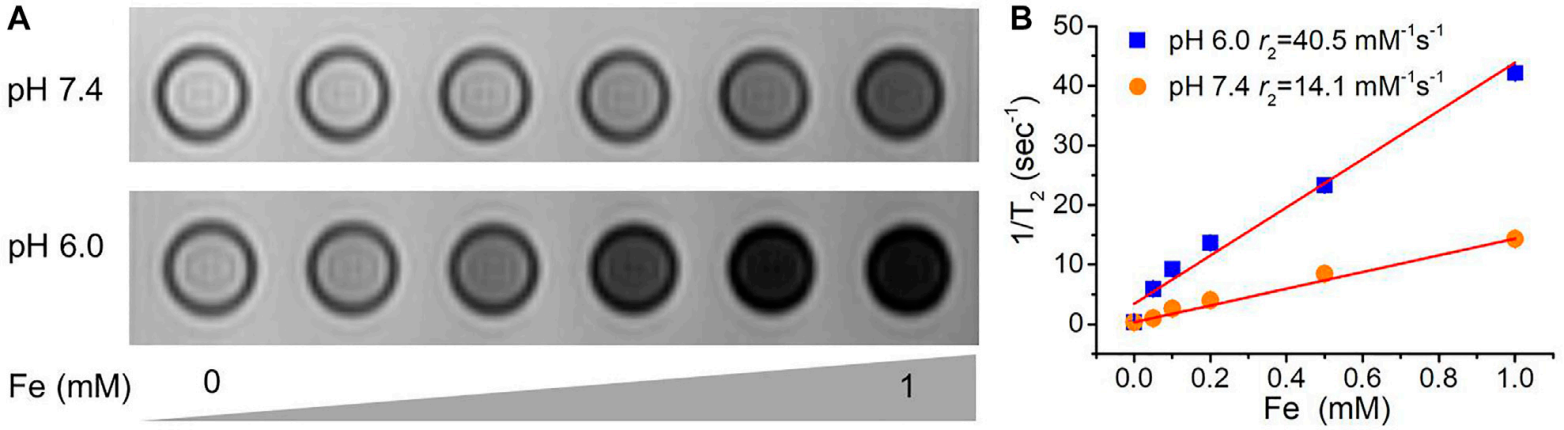
**(A)** T_2_-weighted MR images of the supernatants obtained from Fe/Ce-MSN NPs in FBS solution at different pH values (7.4 and 6.0). **(B)** The *r*_2_ values from T_2_-weighted MR images of Fe/Ce-MSN NPs incubated in above condition.

Such a triggered biodegradation behavior of Fe/Ce-MSN NPs could exert specific functions for theranostics applications. Paramagnetic Fe^3+^ ions have five unpaired electrons with a high magnetic moment, thereby serving as T_2_ contrast agents in MRI (Ahmadov et al., 2015). The T_2_ contrast-enhanced MRI capability of Fe/Ce-MSN NPs was evaluated in an FBS solution with different pHs (7.4 and 6.0) by a clinical 3.0 T human clinical scanner. The significant concentration-dependent darkening effect of Fe/Ce-MSN NP samples was found in T_2_-MR images at a pH of 6.0, whereas the nanoparticle signals in the neutral buffer solution appeared much weaker (Figure 4A). Additionally, the initial transverse relaxivity *r*_2_ of Fe/Ce-MSN NPs at a pH of 6.0 is 40.5 mM^−1^s^−1^, which is much higher than that of the neutral condition samples (pH: 7.4 is 14.1 mM^−1^s^−1^) after incubation for 24 h (Figure 4B). Such contrast-enhanced responsiveness was mainly attributed to a large amount of released Fe^3+^ ions from Fe/Ce-MSNs NPs. Consequently, Fe/Ce-MSN NPs can be regarded as responsive MRI contrast agents in the acidic microenvironment.

### Cellular uptake and anti-oxidative stress of Fe/Ce-MSN-PEG NPs

**FIGURE 5:**
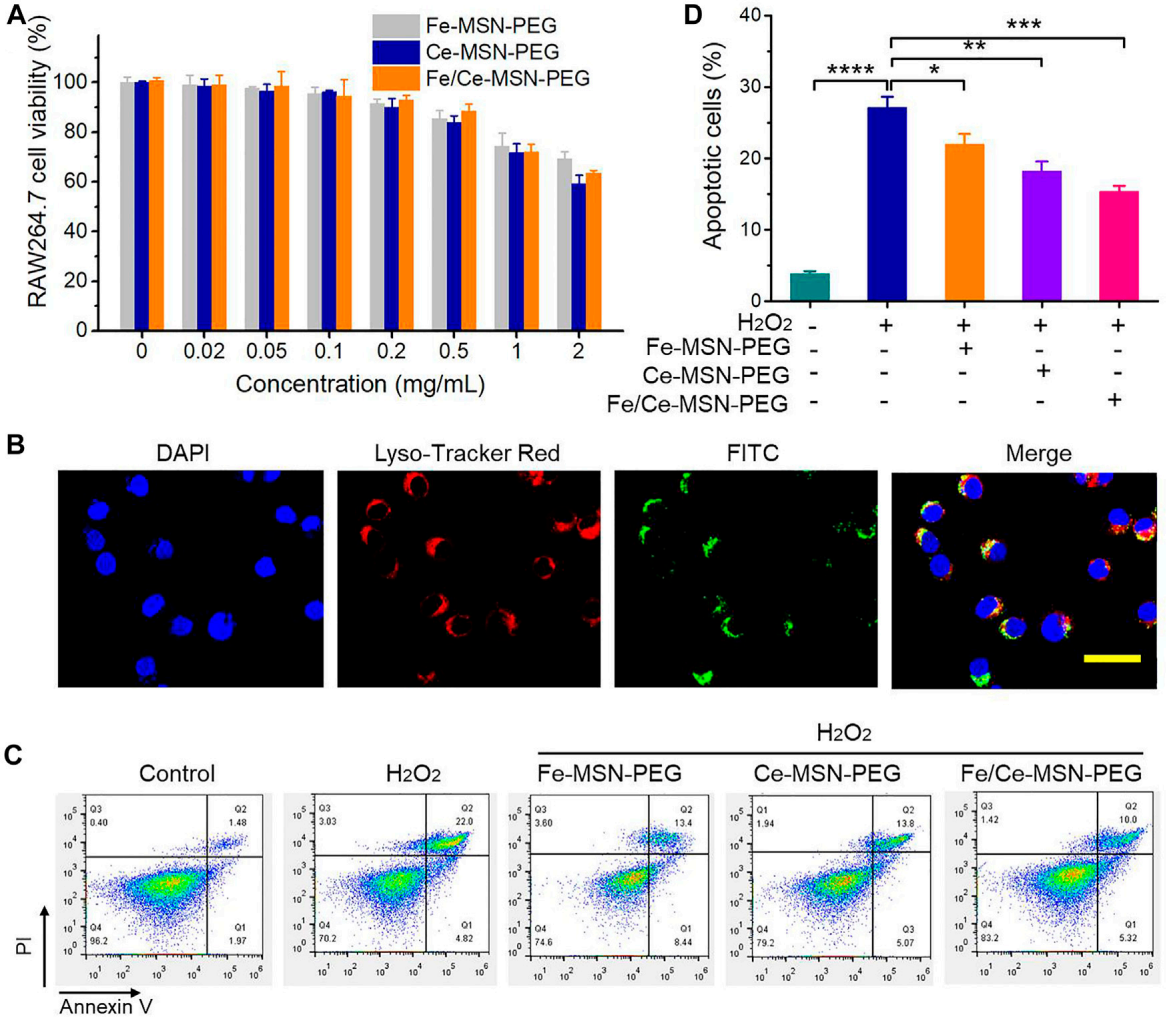
Vitro cytotoxicity, cellular uptake, and anti-oxidative stress of Fe/Ce-MSN-PEG NPs in RAW 264.7 cells. **(A)** *In vitro* cell viabilities of RAW 264.7 cells under different treatment conditions. **(B)** Confocal microscopy observed of the cellular uptake of NPs in RAW 264.7 cells. Nuclei were stained with DAPI (blue), lysosomal were stained with Lyso-Tracker Red (red), while NPs were labeled with FITC (green) (At scale 10 µm). **(C,D)** Flow cytometric profiles and quantitative data of apoptotic RAW 264.7 cells after different treatments. Data are presented as mean ± SE (*n* = 3). *p < 0.05, **p < 0.01, ***p < 0.001.

We first examined the cellular toxicity of nanoparticles (Fe-MSN-PEG, Ce-MSN-PEG, and Fe/Ce-MSN-PEG) in RAW264.7 mouse macrophages and 3T3 fibroblasts before *in vitro* biological evaluations. Relatively high cell viability values were detected after Fe-MSN-PEG, Ce-MSN-PEG, and Fe/Ce-MSN-PEG NPs incubation at varying doses for 48 h. The percentage of viable cells remained above 90% even at Fe/Ce-MSN-PEG NPs of 0.5 mg/ml (Figure 5A). These results indicated that Fe/Ce-MSN-PEG NPs exhibit low cytotoxicity in RAW264.7 mouse macrophages. The toxicity of these nanoparticles to 3T3 fibroblasts was similar to that of macrophages as shown in Supplementary Figure S2A. Then, the cellular uptake of these nanoparticles, which are labeled with a green fluorescence dye (FITC), was determined. The fluorescence image showed that the FITC-labeled NPs were efficiently uptake by RAW264.7 cells after 4 h of incubation, and the green fluorescence signals were mainly located in the cytoplasm (Figure 5B; Supplementary Figure S2B). We then investigated whether Fe-MSN-PEG, Ce-MSN-PEG, and Fe/Ce-MSN-PEG NPs are capable of protecting macrophages from ROS-induced apoptosis. RAW264.7 cell exposure to 200 × 10^−6^ M H_2_O_2_ resulted in significant apoptosis compared with the control cells treated with fresh medium, as illustrated by flow cytometric analysis (Figures 5C,D). In contrast to the three groups, treatment with 0.5 mg/ml Fe/Ce-MSN-PEG NPs significantly attenuated H_2_O_2_-mediated cell apoptosis. The incubation of unstimulating RAW264.7 cells with Fe-MSN-PEG, Ce-MSN-PEG, and Fe/Ce-MSN-PEG did not cause significant apoptosis, which is consistent with the cytotoxicity experiment results, as shown in Supplementary Figures S2C, D. Consequently, only Fe/Ce-MSN-PEG NPs can significantly inhibit oxidative stress-mediated cell apoptosis by effectively scavenging ROS.

### ROS-scavenging activity of Fe/Ce-MSN-PEG NPs *in vitro*

**FIGURE 6:**
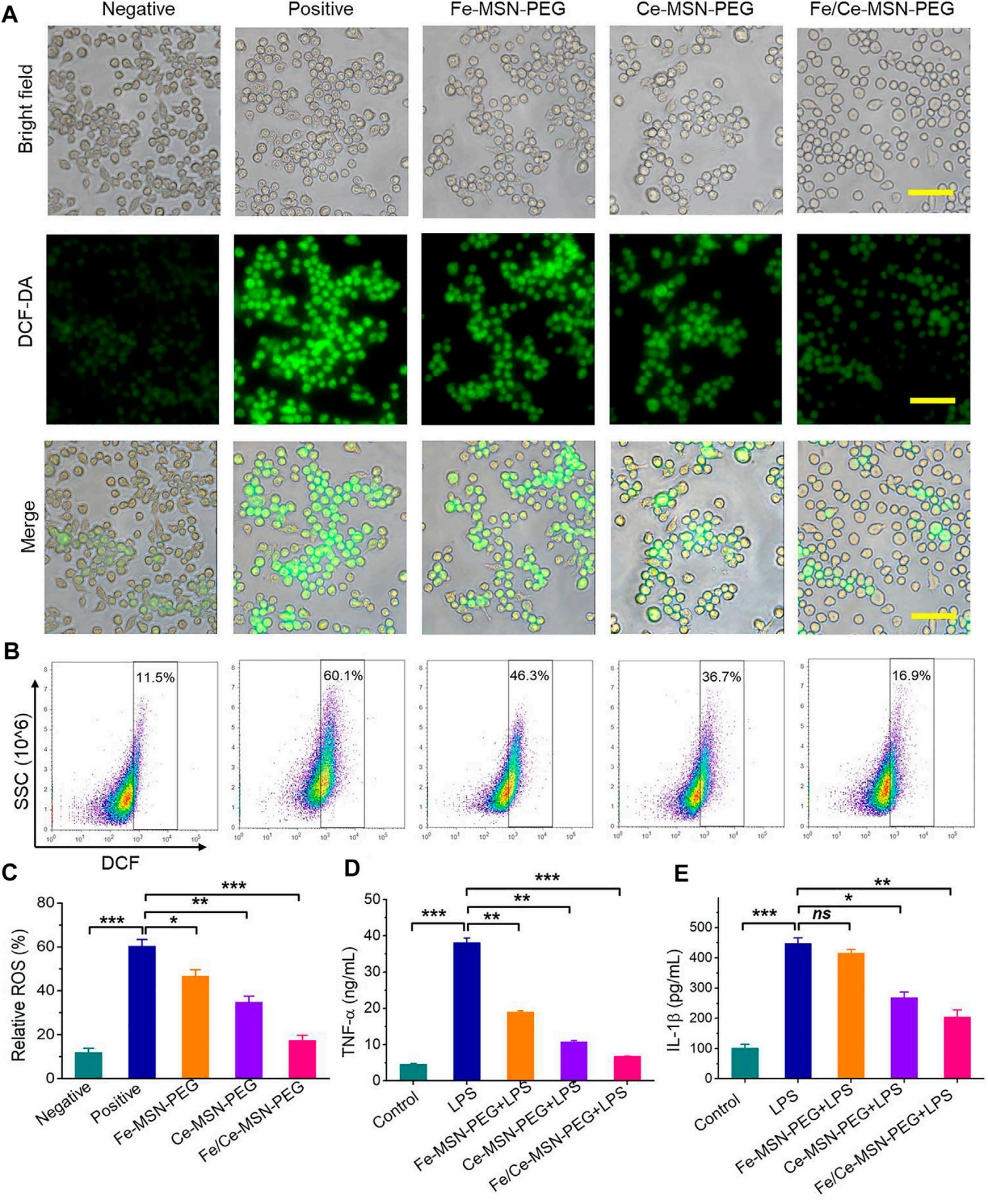
ROS-scavenging and anti-inflammatory activity of Fe/Ce-MSN-PEG NPs *in vitro*. **(A–C)** Representative ROS staining and quantitative data of RAW264.7 cells under different treatment conditions incubated with 200 µM H_2_O_2_. Scale bar: 50 μm. The levels of **(D)** TNF-α, **(E)** IL-1β. Data are presented as mean ± SE (*n* = 3). *p < 0.05, **p < 0.01, ***p < 0.001; ns, no significance.

Additionally, we examined whether Fe-MSN-PEG, Ce-MSN-PEG, and Fe/Ce-MSN-PEG NPs could inhibit the ROS generation in RAW264.7 cells. RAW264.7 cells were treated with 200 µM H_2_O_2_ in the model group, while cells cultured with medium alone served as the normal control (Wang et al., 2015). As probed by a fluorescent dye 2′,7′-dichlorofluorescein-diacetate (DCF-DA) that emits green fluorescence under oxidative conditions, cells in the model group displayed a considerably high ROS level, this after the 4 h stimulation and free-medium incubation for an additional 2 h (Figure 6A). By contrast, fluorescent signals of DCF-DA were significantly attenuated in the Fe/Ce-MSN-PEG NPs group after treating the stimulated RAW264.7 cells with Fe-MSN-PEG, Ce-MSN-PEG, and Fe/Ce-MSN-PEG NPs at doses of 0.5 mgml^−1^ for 12 h. Further quantitative analysis by flow cytometry also demonstrated that intracellular ROS production in stimulated macrophages could be effectively suppressed by Fe/Ce-MSN-PEG NP treatment (Figures 6B,C). Thus, Fe/Ce-MSN-PEG NPs significantly attenuated ROS-induced cell apoptosis in macrophages by eliminating overproduced intracellular ROS.

### Anti-inflammatory activities of Fe/Ce-MSN-PEG NPs *in vitro*

We also interrogated whether Fe-MSN-PEG, Ce-MSN-PEG, and Fe/Ce-MSN-PEG NPs can attenuate inflammatory responses in macrophages. Lipopolysaccharide (LPS)-treated cells expressed a significantly increased excretion of pro-inflammatory cytokines, such as TNF-α and IL-1β, compared to the normal RAW264.7 cells (Figures 6D,E) (Li et al., 2019). Pre-incubation with 0.5 mg/ml of Fe/Ce-MSN-PEG NP for 4 h significantly inhibited the expression of these cytokines. Furthermore, Fe/Ce-MSN-PEG NPs were more effective than Fe-MSN-PEG and Ce-MSN-PEG NPs. Previous findings showed that ROS can activate multiple signal transduction cascades, which in turn modulate inflammation (Münzel et al., 2017). Consequently, our results substantiated that Fe/Ce-MSN-PEG NPs can attenuate inflammation in macrophages by decreasing intracellular ROS production.

### Phenotypic switching of macrophages regulated by Fe/Ce-MSN-PEG NPs

**FIGURE 7:**
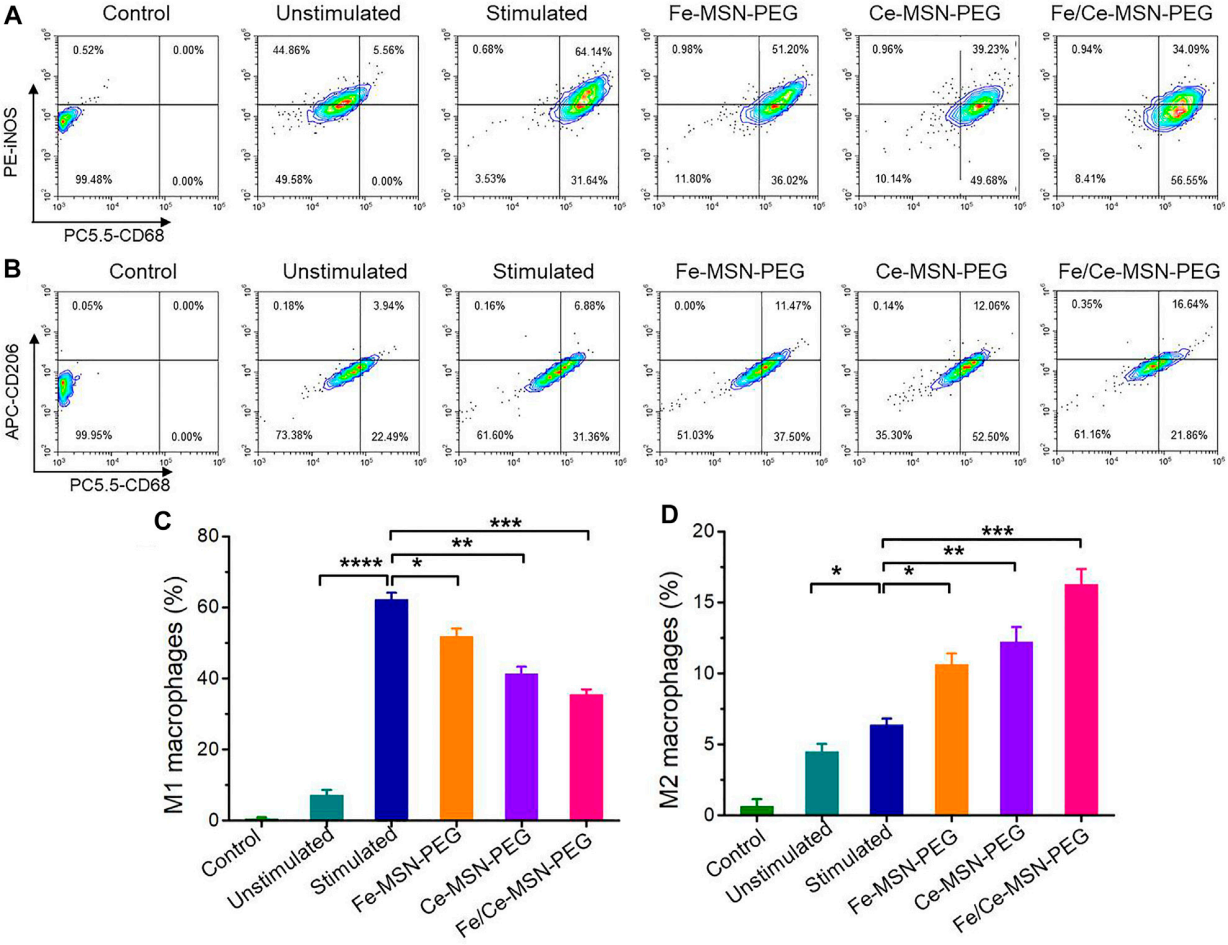
Fe/Ce-MSN-PEG NPs regulates phenotypic switching of macrophages *in vitro*. RAW264.7 macrophages were stimulated with 200 ng ml^−1^ LPS for 24 h, and then treated with various formulations for 8 h. Flow cytometric analysis illustrating the number of **(A,C)** M1 (CD68 + iNOS+) and **(B,D)** M2 (CD68 + CD206+) macrophages. Data are presented as mean ± SE (*n* = 3). *p < 0.05, **p < 0.01, ***p < 0.001.

We further examined the effects of Fe-MSN-PEG, Ce-MSN-PEG, and Fe/Ce-MSN-PEG NPs on macrophage polarization. For RAW264.7 macrophages, treatment with LPS induced a strong pro-inflammatory M1 phenotype, as shown by the significantly increased number of CD68 + iNOS + cells (Figures 7A,C), which is consistent with other literature reports (Guo et al., 2022). After incubation with Fe-MSN-PEG, Ce-MSN-PEG, and Fe/Ce-MSN-PEG NPs, the expression of the M1 biomarker iNOS significantly decreased compared to that of the untreated control. Fe/Ce-MSN-PEG NPs treatment remarkably increased the expression of CD206, a biomarker of the M2 phenotype (Figures 7B,D). Both Fe-MSN-PEG and Ce-MSN-PEG NPs also promoted macrophage polarization, but slightly worse than that of the Fe/Ce-MSN-PEG NPs. These results suggested that Fe/Ce-MSN-PEG NPs promoted the macrophage phenotypic switching from an inflammatory M1 phenotype (iNOS) to an alternatively activated M2 phenotype (CD206) that is beneficial for anti-inflammatory activities.

## Discussion

Inflammation is a crucial natural protective response to various inciting stimuli, such as traumatic, infectious, toxic, or autoimmune injury. The primary inflammation process is intimately linked to oxidative stress, which can be considered an oxidative response to inflammation, and blocking oxidative stress may be beneficial for reducing inflammation (Li et al., 2018). ROS is considered responsible for causing oxidative stress; therefore, selecting an appropriate antioxidant is important for scavenging ROS. Although nano enzymes lack an artificial switch to reversibly regulate the catalytic capacity, in recent years, enzyme mimetics based on ceria nanoparticles (CeNPs) have been exploited as potential substitutes for natural multi-enzyme mimetic activities, e.g., SOD mimics/CAT mimics to scavenge toxic ROS and reactive nitrogen species (NOS), and peroxidase mimics to catalyze H_2_O_2_ (Naganuma, 2021). However, it has been pointed out that nanozymes lack an artificial switch to reversibly regulate the catalytic capacity. Actually, the catalytic activity of ceria nanoparticles against ROS is determined by several factors (Soh et al., 2017). Typically, the atomic Ce^3+^-to-Ce^4+^ ratio is one of the critical factors for catalytic efficacy (Pirmohamed et al., 2010). The Ce^3+^ sites are known to eliminate O_2_•^−^ *via* SOD-mimetics, and •OH by redox reactions, whereas the Ce^4+^ sites are responsible for the H_2_O_2_ oxidation by CAT-mimetics. Therefore, attaining a high Ce^3+^-to-Ce^4+^ ratio during sample preparation is critical for treating inflammation-related diseases because O_2_•^−^ and •OH are directly causing inflammatory responses and cell death (Valko et al., 2007). Additionally, the rate of regeneration from Ce^4+^ to Ce^3+^ is also crucial because shifting to Ce^3+^ is extremely difficult in the auto-conversion process.

The Ce^3+^concentration, which is the main cause of oxygen vacancies, is important for turning the catalytic activity of CeNPs into bio-applications (Dutta et al., 2006). Recent studies revealed that the Ce^3+^ concentration and oxygen vacancy concentration in nanoceria can be turned by the proper metallic dopants. Doping nanoceria with transition metals, such as Fe, Mn, Co., and Zr, can enhance the oxygen diffusion, thereby facilitating enhance surface reactions. Min Soh et al. prepared Zr^4+^-incorporated ceria nanoparticles that possess a higher Ce^3+^/Ce^4+^ ratio and faster conversion from Ce^4+^ to Ce^3+^ than those exhibited by ceria nanoparticles (Soh et al., 2017). The results Zr^4+^-incorporated ceria nanoparticles worked as a potent ROS scavenger, which is attributed to include a higher Ce^3+^/Ce^4+^ ratio as well as the faster Ce^3+^ regeneration rate in this particles. Qin et al. (2019) reported that a novel amorphous Fe_2_O_3_ modified CeO_2_ catalytic system, which plays the role of the engine to greatly improve the O transfer cycle of Ce^4+^/Ce^3+^, as well as enhance the redox and amphoteric properties of the catalysts.

This study described that ferrite and ceria co-engineered mesoporous silica NPs antioxidant agents. In a consequence explained by the ratio changes of Ce^3+^/Ce^4+^ in nanoparticles after Fe was doped, based on the above-mentioned XPS results, the as-prepared Fe/Ce-MSN nanoparticles exhibited an excellent efficiency of ROS, which is attributed to the improvement of multi-enzyme mimetic activity by including the ferrite ion into Ce-MSNs. The improved Fe/Ce-MSN’s ability to scavenge superoxide radicals is attributed to higher SOD enzyme activity, and the improved hydrogen peroxide consumption capacity can be explained by two aspects: maintaining a higher activity CAT enzyme activity and introducing the catalysis effect of Fe_2_O_3_ by including ferrite ion in nanoparticles. The fast biodegradation of Fe/Ce-MSNs, sensitive to the mild acidic microenvironment of inflammation, and thereby enhancing T_2_-weighted MRI in the inflammation site, can accelerate the release of Fe^3+^ ions. Fe/Ce-MSN-PEG NPs *in vitro* cell models significantly attenuated ROS-induced inflammation and cell apoptosis in macrophages by scavenging overproduced intracellular ROS. Moreover, Fe/Ce-MSN-PEG NPs effectively switched macrophages from a pro-inflammatory M1 phenotype toward an anti-inflammatory M2 phenotype, which further reduced expression of pro-inflammatory cytokines TNF-α and IL-1β.

## Conclusion

In summary, we engineered a pH-responsive theranostic nanoplatform of Fe/Ce-MSN-PEG nanoparticle, which exhibits a SOD/CAT-mimetic, that is, capable of eliminating a broad spectrum of ROS. Cellularly, Fe/Ce-MSN-PEG nanotherapy protected macrophages from oxidative stress-induced apoptosis, after internalization into cells and efficiently scavenged ROS, as well as attenuated inflammatory response. These pharmacological activities of Fe/Ce-MSN-PEG NP can facilitate the development of a new type of anti-inflammatory nanotherapies.

## Data Availability

The original contributions presented in the study are included in the article/Supplementary Material, further inquiries can be directed to the corresponding authors.
