# Supplementary material for: pH-responsive theranostic nanoplatform of ferrite and ceria co-engineered nanoparticles for anti-inflammatory
Source: Front Bioeng Biotechnol. 2022 Sep 9;10:983677. doi: 10.3389/fbioe.2022.983677 (PMC9500451; doi:10.3389/fbioe.2022.983677)
Supplement: Supplementary file 1 [file DataSheet1.docx]

Supplementary Information

pH-Responsive Theranostic Nanoplatform of Ferrite and Ceria Co-engineered Nanoparticles for Anti-Inflammatory

Yuanyao Dou ^1,2^, Yimin Zhang ^1^, Caiyu Lin ^1^, Rui Han ^1^,Yubo Wang ^1^, Di Wu ^1^, Jie Zheng ^1^, Conghua Lu ^1^, Liling Tang ^2,^^[[1]](#footnote-1)^* and Yong He ^1,^^[[2]](#footnote-2)^*

1 Department of Respiratory Disease, Daping Hospital, Army Medical University, Chongqing 400042, China

2 Key Laboratory of Biorheological Science and Technology, Ministry of Education, College of Bioengineering, Chongqing University, Chongqing 400044, China


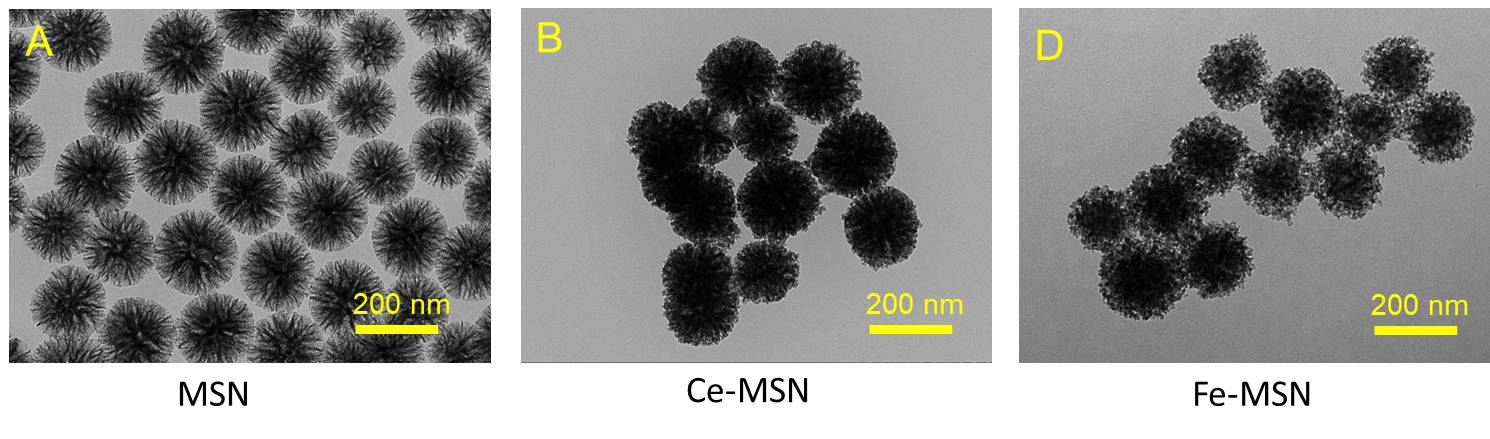


**Figure S1** Morphological and compositional characterizations of Fe-MSN, Ce-MSN NPs: a-c) TEM images of MSN, Fe-MSN, and Ce-MSN NPs.

**
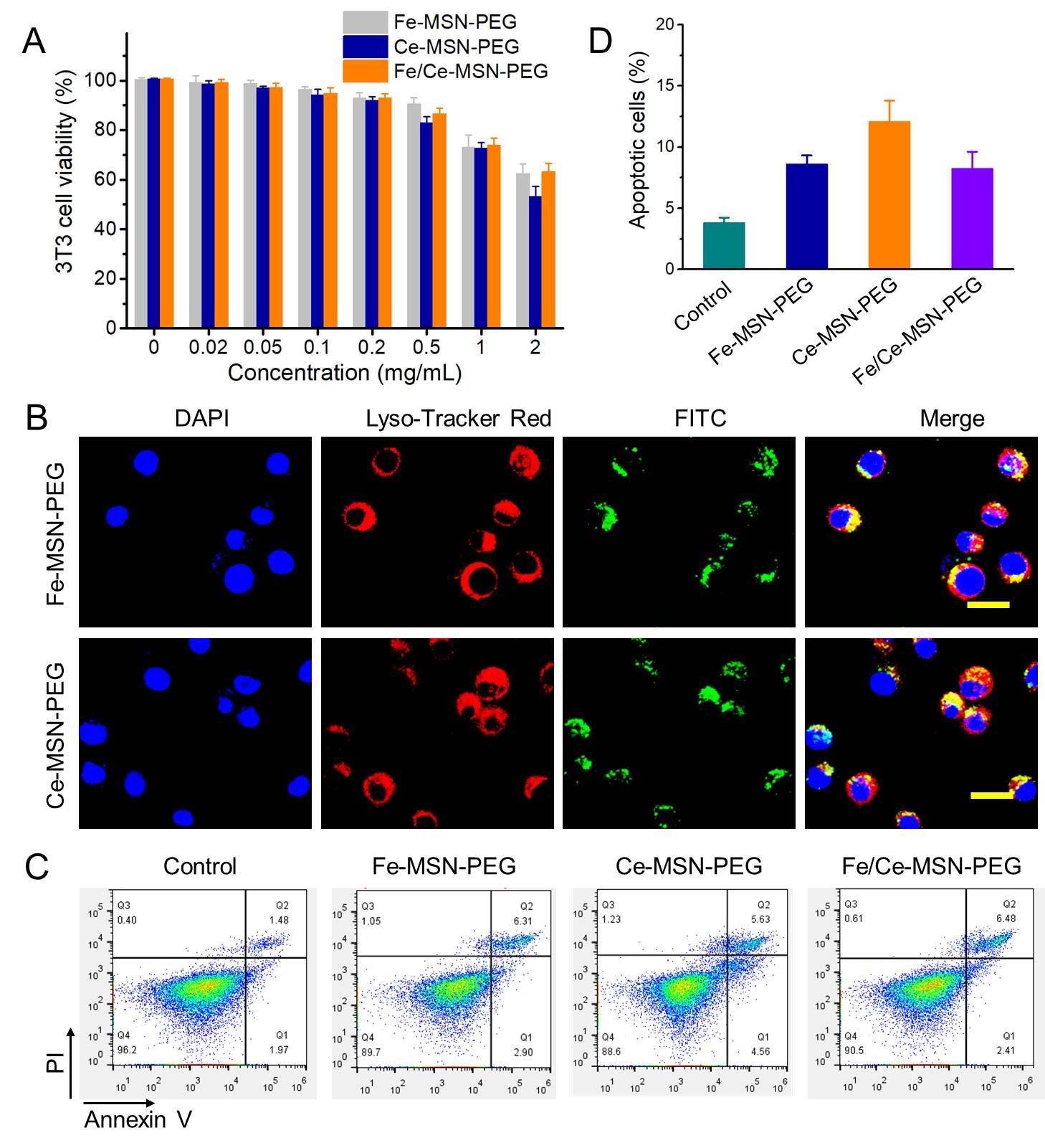
**

**Figure S2** Vitro cytotoxicity, Cellular uptake of Fe-MSN-PEG, Ce-MSN-PEG NPs. a) In vitro cell viabilities of 3T3 cells under different treatment conditions. b) Confocal microscopy observed of the cellular uptake of Fe-MSN-PEG, Ce-MSN-PEG NPs in RAW 264.7 cells. Nuclei were stained with DAPI (blue), lysosomal were stained with Lyso-Tracker Red (red), while NPs were labeled with FITC (green) (At scale 5 µm). c, d) Flow cytometric profiles and quantitative data of apoptotic RAW 264.7 cells after treatment with Fe-MSN-PEG, Ce-MSN-PEG NPs only.

1. *Correspondence: Liling Tang, Key Laboratory of Biorheological Science and Technology, Ministry of Education, College of Bioengineering, Chongqing University, Chongqing 400044, China, Phone: +86-1399-605-1730 E-mail:[tangliling@cqu.edu.cn](mailto:tangliling@cqu.edu.cn). [↑](#footnote-ref-1)
2. Yong He, Department of Respiratory Disease, Daping Hospital, Army Medical University, Chongqing 400042, China. Phone: 86-23-68729084; Fax: 86-23-68729084; E-mail: [heyong@tmmu.edu.cn.](mailto:heyong@tmmu.edu.cn.) [↑](#footnote-ref-2)
